# Immunological Techniques to Assess Protein Thiol Redox State: Opportunities, Challenges and Solutions

**DOI:** 10.3390/antiox9040315

**Published:** 2020-04-15

**Authors:** James Nathan Cobley, Holger Husi

**Affiliations:** Centre for Health Sciences, University of the Highlands and Islands, Inverness IV2 3JH, UK; holger.husi@uhi.ac.uk

**Keywords:** protein thiols, click PEGylation, click chemistry, redox signaling, reactive oxygen species, oxidative stress

## Abstract

To understand oxidative stress, antioxidant defense, and redox signaling in health and disease it is essential to assess protein thiol redox state. Protein thiol redox state is seldom assessed immunologically because of the inability to distinguish reduced and reversibly oxidized thiols by Western blotting. An underappreciated opportunity exists to use Click PEGylation to realize the transformative power of simple, time and cost-efficient immunological techniques. Click PEGylation harnesses selective, bio-orthogonal Click chemistry to separate reduced and reversibly oxidized thiols by selectively ligating a low molecular weight polyethylene glycol moiety to the redox state of interest. The resultant ability to disambiguate reduced and reversibly oxidized species by Western blotting enables Click PEGylation to assess protein thiol redox state. In the present review, to enable investigators to effectively harness immunological techniques to assess protein thiol redox state we critique the chemistry, promise and challenges of Click PEGylation.

## 1. Protein Thiol Redox Biology: An Overview 

Context dependent functionality reconciles the ability of chemically heterogeneous radical and non-radical oxygen, nitrogen, carbon, and sulfur species to be beneficial (e.g., signal) and deleterious (e.g., damage macromolecules) [1,2,3,4,5,6,7,8,9,10]. For example, the ability of reactive species to inflict damage [11,12,13], which was proposed by Denman Harman to cause ageing [14], is exploited by phagocytes to kill ensnared pathogens [15,16,17]. Biological context, therefore, governs whether their immutable chemistry (i.e., set species specific spectrum of chemical reactivity in a biological system) benefits or harms the cell. Or more likely benefits and harms the cell simultaneously to a varying degree (i.e., granularity) set, to a large extent, by the local microenvironment (pH, temperature, solvent accessibility, and vicinal interactome) [18]. Granular context dependent functionality provides a useful theoretical lens to interpret the interplay between reactive species and cysteine residues (i.e., protein thiols). The rich chemical biology of sulfur defined by its ability to occupy eight distinct oxidation states from −2 to +6 affords an apt interface between the heterogeneous thiol proteome and a chemically diverse panoply of reactive species [19,20,21,22,23,24]. The ability of reactive species to interact with the thiol proteome can underlie redox signaling, oxidative damage, and antioxidant defense [25,26,27,28,29,30,31,32,33,34]. None are inherently good or bad: Wayward redox signals have as much potential to cause harm as a quenched sulfur radical in an enzymes active site or antioxidant defense aberrantly silencing or amplifying redox signals. 

Unsurprisingly, given their functional significance, cysteine residues are used parsimoniously (2.26% of the time yields 1 thiol per 50 kDa protein—the mean mass of a typical human protein [35]) and tend to be almost fully conserved or lost completely [36,37]. Additionally, cysteine residues seem to increase with complexity being more prevalent in mammals [38,39]. Analogous to reactive species, treating the ~214,000 cysteines (~50 mM) that comprise the thiol proteome in humans as a homogenous pool is perilous [40,41,42]. Despite possessing the same functional group (i.e., sulfur), the reactivity of protein thiols with hydrogen peroxide (H_2_O_2_) spans at least 6 orders of magnitude [43,44]. Differential kinetics stems from the ability of the local protein environment to deprotonate the thiol (electrostatic gating), stabilize a transition state and/or co-ordinate the leaving group (e.g., by providing a proton relay) [33,45,46,47,48]. For example, the reaction of peroxiredoxin (PRDX) isoforms with H_2_O_2_ (*k* ~10^5^–10^8^ M^−1^ s^−1^) is significantly faster than KEAP1 (*k* ~140 M^−1^ s^−1^) and most thiols (*k* ~10–50 M^−1^ s^−1^) [49,50,51]. Even slow reactions can, however, proceed if they are favored by the local microenvironment and/or facilitated by an enzyme. Transiently inactivating PRDX enzymes could open the floodgates for H_2_O_2_ to signal [52]. Further, PRDX enzymes can transmit redox signals by transferring H_2_O_2_ derived electrons to a target (i.e., a redox relay) [53,54,55,56]. Beyond H_2_O_2_, a role for free radicals (e.g., nitrogen dioxide radical) and other non-radical species (e.g., peroxynitrite) must be considered [57,58]. 

**Figure 1:**
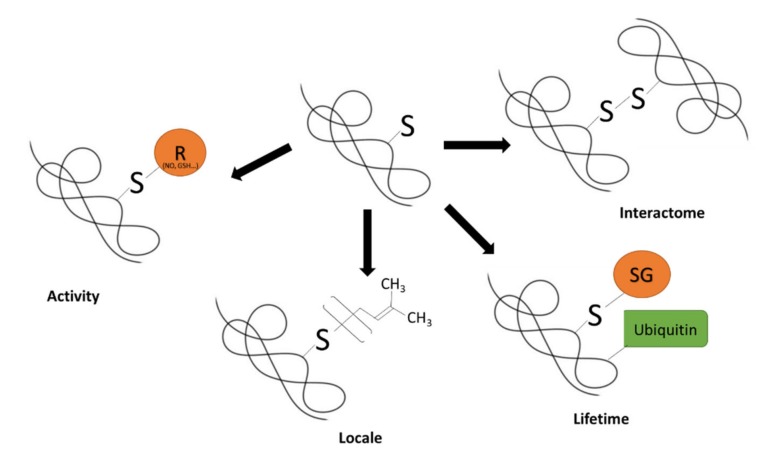
Functional aspects of reversible thiol oxidation. The schematic depicts the four main impacts of reversible thiol oxidation on protein function: activity, locale, interactome, and lifetime. For illustrative purposes, *S*-prenylation is depicted and an RSSG modification leading to ubiquitination. In principle, any modification could exert an effect by any of the major functional aspects described (e.g., interactome effects are not restricted to RSSR).

Regardless of the functional consequences, reactive species interact with the heterogenous thiol proteome by changing sulfur oxidation state via electron exchange. One major outcome is an increase in the amount of a thiol that is reversibly oxidized (i.e., a fractional increase in reversible thiol oxidation occupancy). Thiyl radicals (RS^•^) and sulfenic acids (SOH) define the common starting point for free radical and non-radical reactions, respectively [20,57,59,60]. RS^•^ and SOH provide an initial platform for a rich set of chemically heterogenous modifications with disparate functionality (Table 1) [19,20]. In principle, a shift in the fractional occupancy of a thiol can enact a functional change by altering protein: activity, locale, interactome, and lifetime (Figure 1) [28,61,62]. Moreover, distinct chemical biology means different modifications can exert diametrically opposed effects even when they modify the same thiol. A redox code may exist wherein the biological outcome may differ depending on the reversible oxidation occupancy of constituent protein thiols (i.e., a shift in one thiol may tip the balance towards a given outcome) [63]. The fractional reversible thiol occupancy is dynamic: it shifts as a function of differences in the rate of formation and removal over time [64]. For example, a change in NADPH metabolism able to decrease peroxidase mediated H_2_O_2_ metabolism would suffice to increase reversible thiol oxidation occupancy even if the rate of formation stayed constant. Ultimately, residing at the strategic nexus of oxidative stress, antioxidant defense, and redox signaling the thiol proteome is central to understanding the biological role of reactive species in health and disease across the lifespan from development to ageing. 

**Table 1:** Major reversible thiol modifications by type. Key reactions and enzyme regulated, and selected examples are provided. Note many more important modifications (e.g., *S*-acetylation [65]) exist. The table merely provides a brief overview of some of the key modifications.

Modification	Example Reaction	Enzyme Regulation	Selected Examples
Sulfenic acid (SOH)	RS^−^ + H_2_O_2_ → RSOH + H_2_O	Thioredoxin/PRDX.	EGF receptor SOH at Cys797 potentates tyrosine kinase activity [66]. Src Cys185 and 277 SOH enhances protein activity [67].
Thiyl radical (RS^•^)	RS^−^ + NO_2_^•^ → RS^•^ + NO_2_^−^	n/a	RS^.^ play a role in the reversible oxidation of thiols to RSSG in NDUFV1 and NDUFS1 in complex I [68], which can inhibit enzyme activity [69,70,71,72,73]. A catalytic RS^.^ enables ribonucleotide reductase to remove an oxygen atom from the 2-OH position in ribose to yield deoxyribose [74].
Disulfide bonds (RSSR)	RSOH + RSH → RSSR + H_2_O	Thioredoxin isoforms.	Intermolecular RSSR moieties activate ATM dimers to initiate DNA double strand break repair [75]. RSSR exchange between PRDX2 and STAT3 represses STAT3 transcriptional activity [53]. RSSR of plasma membrane bound HPLAC1 enables plants to sense and respond to extracellular H_2_O_2_ [76].
Glutathionylation (RSSG)	RS^−^ + GSSG → RSSG + GS^−^	Glutaredoxin isoforms.	eNOS RSSG at multiple sites enhances uncoupling mediated superoxide production [77]. SMYD2 Cys13 RSSG dissociates SYMD2 from titin leading to sarcomere instability [78].
*S*-nitrosation (RSNO)	RS^•^ + NO^•^ → RSNO	Protein mediated NO^•^ transfer.	ND3 Cys39 RNSO holds complex I inactive to prevent oxidative damage in ischemia reperfusion injury [79,80,81]. LURE1 RSNO inhibits polyspermy in flowering plants [82].

## 2. The Case for Using Immunological Techniques to Assess Protein Thiol Redox State 

The functional significance of many protein thiols reinforces the importance of assessing their redox state. Protein thiol redox state is usually assessed using redox proteomics approaches [83,84,85,86,87,88,89]. Redox proteomic affords hypothesis-free, systematic, and quantitative global thiol proteome profiling [90]. For example, state-of-the-art cysteine reactive phosphate tag technology coupled to tandem mass tag multiplexing identified and quantified 171,000 thiols modification events in mammalian tissues [91] (i.e., ~80% of the total thiol proteome was assessed). Low-throughout immunological techniques (e.g., ~2 thiols per experiment or 0.0009% of the human thiol proteome) may seem redundant when one can quantify the redox state of thousands of protein thiols in parallel in a single experiment. Analogous to Western blotting in standard proteomics [92], the value of immunological techniques stems, in part, from their ability to verify redox proteomic findings. Verifying redox proteomic findings using a complementary orthogonal technique enables protein identity to be immunologically confirmed as opposed to being assigned based on a unique peptide mass alone [93,94]. Quantifying each modified thiol relative to the entire protein is important because peptide analysis could be biased (e.g., some may be unsuitable for electron spray ionization). Further, studying each thiol enriches redox proteomic findings by placing the original finding in context. Target redox state context is important for concluding whether the protein *per se* (i.e., all target thiols) or an individual thiol responds to given stimuli/context (e.g., cardiovascular disease [95]). Without immunological analysis one could conclude a single thiol is reversibly oxidized in cardiovascular disease when all target thiols are. Far from being trivial, such nuances can have profound consequences for interpreting how key biological phenomena impact the thiol proteome and for developing biomarkers. Ideally, immunological assays would be performed in parallel with targeted multiple reaction monitoring (MRM) to identify the thiols (i.e., sites) modified [96,97]. 

The value of immunological techniques extends well beyond merely verifying redox proteomics findings. In many cases, immunological techniques represent the only viable way to assess certain targets. For example, redox proteomics studies often fail to detect hydrophobic protein thiols [98]. Even state-of-the-art cysteine reactive phosphate tag technology was unable to detect two hydrophobic complex I subunits (i.e., ND6 and ND4L [91,99]). Their hydrophobicity makes proteomics, yet alone redox proteomics, challenging [100]. Moreover, certain thiols remain unstudied because they form part of linear amino acids sequences recalcitrant to tryptic digestion. As Held [89] remarks, recalcitrance to tryptic digestion often precludes analysis of the active site thiol (Cys215) in PTP1B. Additionally, data dependent acquisition (DDA) presents difficulties for detecting thiols on low abundance proteins [90]. In DDA, low abundant thiols are effectively masked by highly abundant peptides preferentially fragmented to daughter ions in MS-MS. Immunological techniques are, therefore, required to detect many protein thiols. Above all, immunological techniques open-up new opportunities to study the thiol proteome for investigators who lack access to mass spectrometric facilities. Even when mass spectrometric facilities are available, the cost and expertise required can preclude redox proteomics. Further, when redox proteomics is possible, access to a complementary orthogonal technique can only enrich the field. Analogous to immuno-spin trapping for electron resonance spectrometry [101], the overarching goal of immunological techniques is to place protein thiol redox biology into the hands of the masses by empowering any investigator to assess the redox state of a target protein using simple, time and cost-efficient methods. 

## 3. Novel Immunological Techniques to Assess Protein Thiol Redox State 

### 3.1. Click PEGylation 

**Figure 2:**
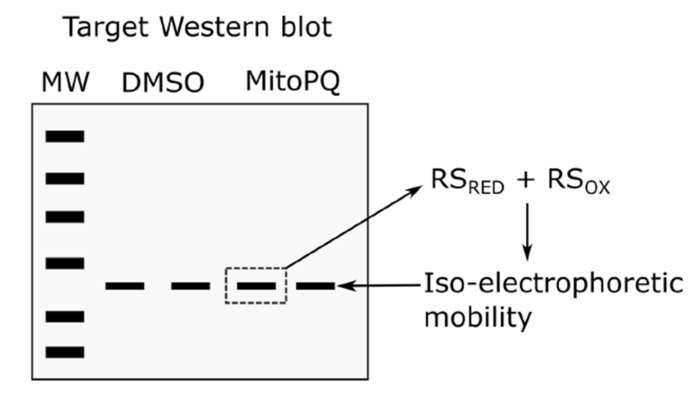
The iso-electrophoretic mobility problem. An exemplar scenario is depicted wherein Western blotting is used to detect a change in the redox state of a target in biological samples treated with and without (i.e., DMSO) the pro-oxidant mitochondria targeted paraquat (MitoPQ). For many protein thiols, Western blotting is unable to detect differences in target redox state because reduced and reversibly oxidized thiols possess similar electrophoretic mobility. MW denotes molecular weight.

Until recently, investigators were unable to harness immunological techniques to assess protein thiol redox state. The inability to distinguish between reduced and reversibly oxidized protein species owing to their iso-electrophoretic mobility rate-limits attempts to assess protein thiol redox state by Western blot (i.e., immunologically). Unless reversible thiol oxidation alters oligomeric state (e.g., typical 2-Cys PRDX isoforms dimerize when they form intermolecular disulfide bonds [102,103,104]), then Western blotting is unlikely to report protein thiol redox state. For example, one would be unable to identify whether selectively inducing superoxide at the flavin mononucleotide group in complex I using mitochondria-targeted paraquat [105,106] suffices to reversibly oxidize a given target using Western blotting (Figure 2), which limits understanding of the sources and targets of mitochondrial superoxide [107,108,109]. Even when a change in oligomeric state makes Western blotting possible, it is often orthogonal to certain thiols and modifications. For example, in the typical 2-Cys PRDX dimer assay RSSG species could be present in the “reduced” monomeric band and the redox state of additional thiols (i.e., beyond the RSSR pair) will be missed. Moreover, unless target immunocapture is performed, SOH, RSNO, and RSSG blotting alone (e.g., avidin detection of biotinylated glutathione labelled samples [110]) fails to disclose the proteins modified and are limited by quantification issues (e.g., loading controls) and inability to resolve individual thiol modifications (i.e., number of RSSG sites). Techniques like biotinylated glutathione are, however, invaluable for global modification screening and purifying samples for redox proteomics [110]. 

To detect reversibly oxidized thiols with iso-electrophoretic mobility by Western blotting, one must ectopically achieve a mobility shift by ligating a bulky biocompatible moiety to a thiol. In 2013, Eaton’s group built on the biotin switch assay by ligating *N*-ethylmaleimide (NEM) functionalized polyethylene glycol (PEG) to mobility shift reversibly oxidized protein thiols, termed the PEG switch assay [111]. NEM functionalized PEG (mPEG) has proven invaluable for assessing protein thiol oxidation. For example, Murphy’s group [112] used mPEG to show that mitochondrial reactive species inactivate pyruvate dehydrogenase kinase 2. Recently, Jakob’s group [113] demonstrated that histone methyltransferase oxidation, evidenced using mPEG, decreases methylation, which enables stochastic fluxes in developmental reactive species to regulate lifespan via an epigenetic switch. The utility of mPEG is, however, limited because the bulky PEG sterically impedes thiol labelling [98]. To solve the steric problem, the Cochemé group used copper (I) catalyzed azide-alkyne Click (CuAAC) to split the labelling reaction into two steps [98]. A sterically free labelling step is achieved by alkylating protein thiols with a heterobifunctional alkyne maleimide reagent. CuAAC is deployed to conjugate alkyne labelled thiols to azide functionalized PEG via a stable triazole [114]. The necessity for a cytotoxic catalyst is, however, problematic [115]. We extended their work by using Inverse Electron Demand Diels Alder (IEDDA) chemistry to develop catalyst-free Click PEGylation workflows [116]. The following subsections critique the chemistry, promise, and challenges of catalyst-free Click PEGylation. 

### 3.2. Underlying Principles 

**Figure 3:**
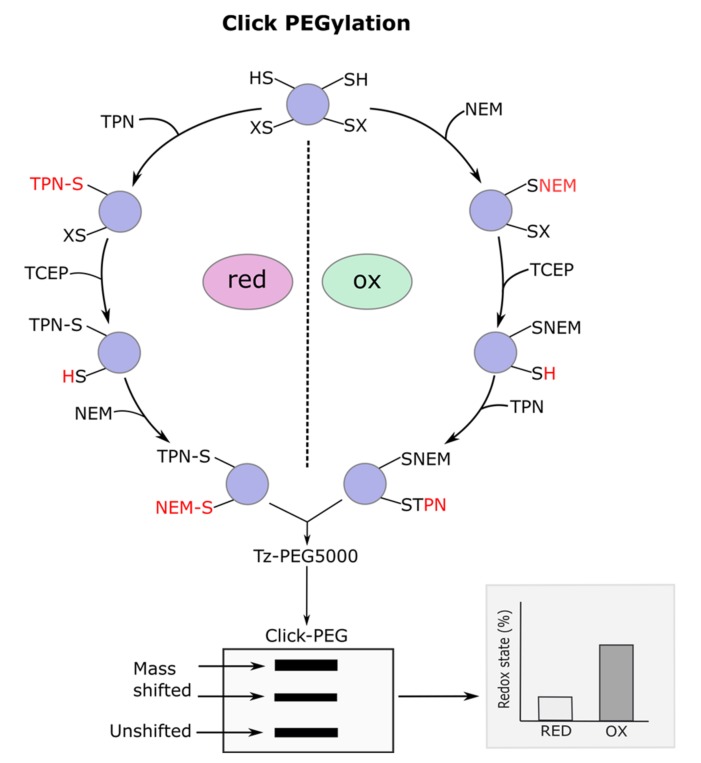
Catalyst-free Click PEGylation schematic. Modified with permission from Cobley et al [116]. The left side of the circle depicts the Click PEGylation reduction (Click-PEGRED) protocol wherein reduced thiols are labelled with TCO- polyethylene glycol 3 (PEG)-maleimide (TPN); (2) before 6-methyltetrazine PEG 5 kDa (Tz-PEG5) is added to initiate the IEDDA Click reaction to mass shift reduced thiols. An optional Tris(2-carboxyethyl)phosphine hydrochloride (TCEP) reduction step to reduce reversibly oxidized thiols before labelling them with *N*-ethylmaleimide (NEM) is included. The right side of the circle depicts the Click PEGylation oxidation (Click-PEGOX) protocol wherein (1) reduced thiols are labelled with NEM; (2) reversibly oxidized thiols are reduced with TCEP; (3) before being labelled with TPN; and (4) Tz-PEG5 is added to initiate IEDDA Click reaction to mass shift reversibly oxidized thiols. A subsequent Western blot of a target is depicted wherein the Click PEGylated bands are selectively mass shifted. For example, in the Click-PEGOX workflow the mass shifted bands correspond to reversibly oxidized thiols (each one being shifted by approximately 5 kDa) and the unshifted band corresponds to the reduced protein. Following densitometry, percent reversibly oxidized protein can be quantified (as depicted in the inset).

Catalyst-free Click PEGylation to assess reversibly oxidized thiols involves four key steps (Figure 3). First, reduced protein thiols are alkylated to render them orthogonal to subsequent labelling steps. Typically, NEM is used to form a thioether bond via Michael addition with the sulfhydryl/thiolate [19]. NEM is normally preferred to iodoacetamide (IDA) because it reacts appreciably faster with reduced thiols [20]. Care must be taken to ensure buffer pH is between 6.5 to 7.5 to prevent off-target amino acid labelling (e.g., lysine) [19,20,117]. Likewise, excess unreacted NEM, as well as, glutathione (GSH) and l-cysteine must be removed with a spin column and/or a quenching step to prevent downstream interference. Second, reversibly oxidized thiols are reduced with a generic chemical reductant like Tris(2-carboxyethyl)phosphine hydrochloride (TCEP) or 1,4-Dithiothreitol (DTT). TCEP is preferred because DTT can autoxidize to produce superoxide when transition metal ion (e.g., Fe^2+^) catalysts are present (as is likely the case in complex biological samples) [74]. Additionally, commercially available TCEP reducing gels omit the need for a second spin column step. Optionally, TCEP/DTT can be substituted for selective reductants (e.g., copper and ascorbate for RSNO [118]) to unveil reversible modification type. The ability to disclose the number of thiols modified by a particular chemotype (e.g., RSNO) represents a key advantage of Click PEGylation compared to other immunological techniques (e.g., target immunocapture followed by RSNO antibody probing). 

Third, newly reduced (i.e., reversibly oxidized) thiols are alkylated with *trans*-cyclooctene (TCO)-PEG3-maleimide (TPN) via Michael addition. The short PEG3 linker enhances TPN hydrophilicity, flexibility, and accessibility [116]. Excess TPN should be removed via a spin column to prevent unproductive competition with TPN decorated protein thiols. Care should be taken when storing TPN to prevent unclickable TCO isomers forming [119,120]. Fourth, TCO labelled thiols are PEGylated by adding 6-methyltetrazine functionalized PEG 5000 (Tz-PEG5) to achieve a 5 kDa mobility shift per modified thiol via IEDDA, which renders reversibly oxidized thiols detectable by Western blotting. Methyl substituted tetrazines are preferred owing to their enhanced stability [120]. Reciprocal reduced protein thiol analysis can be performed by starting at step 3. Mass shifted band intensity is internally normalized to unshifted band (i.e., reduced) intensity to calculate percent reversible thiol oxidation. No between-lane loading control is required because data are internally normalized [116]. Additionally, the contribution of each mass shifted band (i.e., each individual thiol) to the total reversible oxidized signal can be calculated. Click PEGylation, therefore, enables reversible thiol oxidation to be quantified by Western blotting [98,116]. Importantly, Click PEGylation is compatible with cell, isolated organelle, and tissue lysate analysis. 

Redox biologists will be familiar with Diels Alder chemistry because it enables singlet dioxygen to react with unsaturated fatty acids to initiate lipid peroxidation [121]. IEDDA involves an electron rich dienophile reacting with an electron deficient diene to yield a stable 4,5-dihydropyrazine conjugate in the absence of a catalyst [122,123,124]. As Oliveira and colleagues remark [124], 1,4 addition of 6-methyltetrazine diene to the TCO alkene yields a strained catalytic intermediate able to evolve to 4,5-dihydropyrazine after releasing nitrogen (i.e., a benign byproduct). IEDDA mediated Click PEGylation confers three important advantages: (1) it obviates the need for a cytotoxic copper catalyst; (2) it affords selective, bio-orthogonal conjugation; and (3) enables rapid conjugation since IEDDA proceeds 10,000 times faster than CuAAC [122,123,124]. Table 2 summarizes the key advantages and disadvantages of Click PEGylation. In principle, therefore, IEDDA mediated Click PEGylation enables one to immunologically quantify protein thiol redox state by Western blotting. 

### 3.3. Click PEGylation Is A Useful Tool to Assess Protein Thiol Redox State: A Mitochondrial ATP Synthase Case Study

Recent studies substantiate the ability of Click PEGylation to assess protein thiol redox state (e.g., [98]). To highlight the advantages of Click PEGylation, we consider the mitochondrial ATP synthase (i.e., complex V). The mitochondrial ATP synthase harnesses the electrochemical proton motive force across the inner mitochondrial membrane to synthesize ATP from inorganic phosphate and ADP via a catalytic rotary mechanism [125,126,127]. Since the seminal work of Yagi and Hatefi in 1984 [128], it has been appreciated that reversible thiol oxidation can regulate mitochondrial ATP synthase activity. Subsequent studies have unraveled the identity of the reversibly oxidized subunits, as well as, the sites and types of modification [129]. To give a key example, reversible oxidation of the matrix facing ATP synthase F_1_ alpha subunit at two evolutionary conserved thiols (Cys244 and 294) seems to impair catalysis [129,130,131]. 

**Figure 4:**
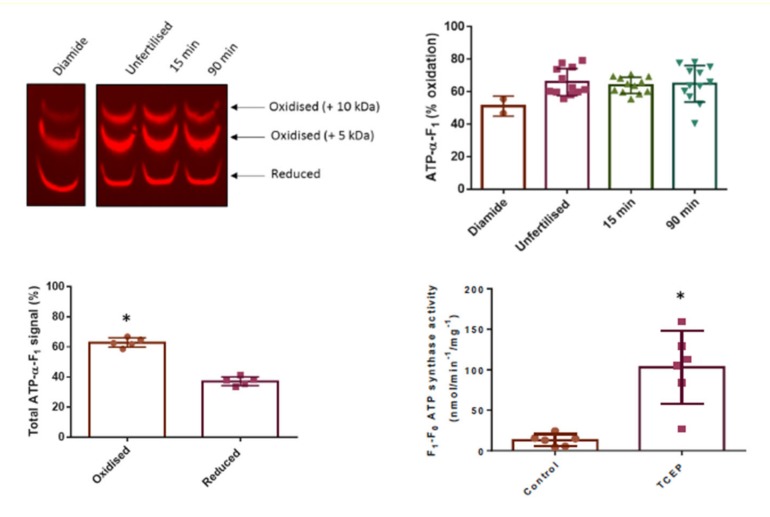
Reversible oxidation regulates mitochondrial ATP synthase activity in *X. laevis* oocytes. Modified with permission from Cobley et al [116,137]. The top right figure shows an illustrative example of a catalyst-free IEDDA Click PEGylation blot against the alpha subunit of the mitochondrial ATP synthase. Quantifying the mass shifts (top left figure) reveals the alpha subunit is substantially oxidized before and after fertilization. The bottom figure shows reversible thiol oxidation of the alpha subunit is statistically significant (denoted by an Asterix) in oocytes. The bottom right figure shows that chemically reducing thiol oxidation significantly (statistical significance is denoted by an Asterix) increases mitochondrial ATP synthase activity in isolated *X. leavis* oocyte mitochondria. Click PEGylation, therefore, helped unveil a regulatory role for reversible thiol oxidation in early development.

Until recently, reversible subunit alpha oxidation had only been assessed by redox proteomics usually in either isolated mitochondria or disease models [131]. Intrigued by a possible physiological redox regulatory role, we asked whether the alpha subunit is reversibly oxidized in oocytes and zygotes from the key developmental model *Xenopus laevis* (*X. laevis*) [132,133,134]. We hypothesized that the alpha subunit would be reversibly oxidized in oocytes to prevent wasteful ATP hydrolysis [135], but that fertilization induced ADP may provide an instructive cue to relieve reversible thiol oxidation to initiate embryonic mitochondrial ATP synthesis [136]. Unexpectedly, reciprocal reduced and reversibly oxidized catalyst-free Click PEGylation workflows revealed the ATP synthase is substantially (~65%) oxidized before and after fertilization (Figure 4) [116]. To place our findings in context, the median oxidation of thiols in the mammalian proteome is ~12% [41] and the Oximouse dataset (see [91]) reveals C244 and C294 are ~20% reversibly oxidized. Substantial reversible thiol oxidation is consistent with a distinct subset of thiols being persistently oxidized (≥20%) and tissue specific redox signatures [91]. Click PEGylation reveals substantial reversible oxidation of ~20% of the total available thiols (~10-11) in the *X. laevis* ATP synthase. Further analysis revealed a single thiol was preferentially modified. Consistent with a single thiol being modified, selective reduction experiments unveiled RSSG as the dominant reversible thiol oxidation type [116]. Reversible thiol oxidation is biologically meaningful because reducing oxidized thiols significantly increased mitochondrial ATP synthase activity in *X. laevis* oocytes (Figure 4) [137]. 

While the identity of the critical redox switch(es) remains elusive (several subunits are reversibly oxidized), Click PEGylation was crucial for unravelling a novel redox regulatory role for reversible ATP synthase oxidation in development [138,139,140]. From a wider theoretical perspective, temporal context may govern the outcome of redox switches in the mitochondrial ATP synthase. Perhaps, they are beneficial in early life (e.g., to constrain ATP hydrolysis) but deleterious in later life when aberrant reversible thiol oxidation compromises mitochondrial ATP synthesis [141]. The mitochondrial ATP synthase example reinforces the ability of Click PEGylation to: (1) test experimental hypotheses; (2) quantify reversible thiol occupancy; (3) quantify the number of thiols modified; (4) assess the relative contribution of each modified thiol to the total reversible oxidation observed; and (5) quantify reversible thiol oxidation type in complex biological samples. 

### 3.4. Challenges

**Figure 5:**
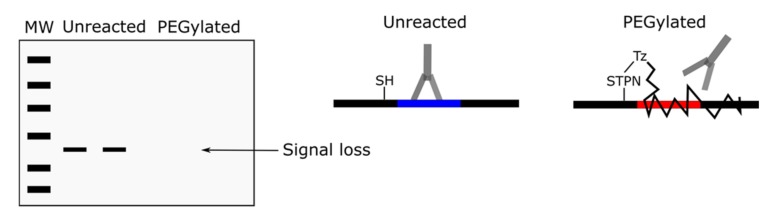
Click PEGylation challenges. Left to right. Signal loss in the PEGylated compared to the unreacted lanes is often observed. In the unreacted samples, the antibody can bind to the epitope. PEGylation, however, may sterically impede epitope binding resulting in partial or complete signal loss.

Beyond the inability to resolve the identity of the thiol oxidized (unless a protein with a lone thiol is assessed; e.g., ND3 Cys39 [142]), antibody binding concerns beset Click PEGylation (Figure 5). Click PEGylation will unilaterally fail to report protein thiol redox state if the antibody is unable to bind the PEGylated protein. The bulky PEG polymer likely sterically impedes antibody binding by physically occupying the epitope [98,116]. Epitope occupancy probability is likely to increase proportional to the number of PEGylated thiols, especially if they are evenly distributed over the linear denatured protein. Even distribution presents difficulties for polyclonal antibodies. Proximity of the PEGylated thiol to the epitope will also influence antibody binding. A protein with a lone thiol distal to a single epitope (i.e., monoclonal) should be amenable to Click PEGylation. Additionally, the probability of epitope occupancy seems to increase when PEGylation adds significant mass to a protein. We have found that PEGylation fails for ND3 (unpublished data) with Tz-PEG5000 (~38% of mass added) but successfully detects ATP synthase subunit alpha (~18% of mass added). Unfortunately, steric hinderance may persist even when smaller PEG moieties (e.g., Tz-PEG2000) are used [98]. Moreover, adding mass to a protein raises the possibility of unequal transfer of PEGylated proteins onto a PVDF membrane leading to the mass shifted signal being underestimated. The unsuitability of many strategically important protein thiols for Click PEGylation rate-limits attempts to study protein thiol redox state using immunological techniques. For example, Lee and Chang found that alkylating reduced thiols with m-PEG5000 led to an inability to detect many strategically important proteins, including glutathione reductase, calcium calmodulin kinase II, and nuclear factor kappa beta [143].

Additional challenges include the ability of SDS to distort PEGylated bands, inability to multiplex by re-probing a blot, and unsuitability for certain species (e.g., *Drosophila melanogaster*) due to a lack of antibodies [116]. While antibodies can be raised against certain species, the biophysical interaction between PEG and SDS is difficult to surmount [144]. Native blotting is an obvious solution but relies on conformational antibody availability [144]. Additionally, native blotting is comparatively time consuming (e.g., transfer time is at least 4 h compared to 1 h for Western blotting [145]). The SDS interference can be minimized by removing excess Tz-PEG5 with a spin column and limiting [SDS] in the Laemmili buffer [116,146]. The inability to multiplex may be overcome by stripping the membrane before re-probing against a new target. Differential protein losses between the total and mass shifted bands could, however, confound the analysis. While multiplexing a single blot is problematic, PEGylated lysates yield enough material to assess multiple proteins [143]. 

Click PEGylation relies on the unshifted total band representing the reduced protein. Analogous to the typical 2-Cys PRDX dimer assay (or any oligomeric shift assay), TCEP/DTT irreducible sulfinic (SO_2_) and sulfonic acid (SO_3_) species will contaminate the reduced unshifted band. SO_2_/SO_3_ occupancy can be partially estimated with reciprocal reduced and reversibly oxidized Click PEGylation [98]. Strategies are required to define their specific occupancy by selective reduction and/or mass shifting reduced and reversibly oxidized thiols together—any unshifted protein should correspond to the irreversibly oxidized form. Anything beyond a binary doublet signifies partial SO_2_/SO_3_ occupancy. Accounting for SO_2_/SO_3_ occupancy is important since recent electrophilic nitrogen species based chemically selective proteomic profiling reveals 387 thiols (e.g., NDUFS1 Cys92) can be oxidized to SO_2_ [147]. Their sensitivity to sulfiredoxin [148] catalyzed reduction means SO_2_ is, in over 50 cases (e.g., Cofilin Cys39), reversible [147]. Reversibility implies an underappreciated role for SO_2_ in redox signaling and antioxidant defense. 

Troublingly, NEM and IDA can react with SOH species [149], which implies reversible thiol oxidation could be underestimated given SOH occurs on more than 1200 thiols distributed across ~700 proteins (e.g., Src kinase Cys185 and 277) [67,150]. Furthermore, NEM and IDA can also react with biologically significant SO_2_ and persulfide species [147,151,152]. The second-order bimolecular reaction between an alkylating agent and SOH is, however, significantly slower than the thiol labelling reaction [149], suggesting it could be mitigated by titrating the molar mass used and reaction time. If methylsulfonyl benzothiazole is used to label reduced thiols without reacting with SOH species, then the ability of the resultant SO_2_ species to distort downstream analysis by reacting with RSNO should be considered [153]. As the development of tunable cysteine reactive phosphate tags attests [91], untuned alkylating agents can fail to label many proteins despite their molar excess. Incomplete labelling likely relates to an inability to tune hydrophobicity to local protein environments (e.g., hydrophobic mitochondrial membrane ensconced proteins). 

A challenge is the ability of destructive analysis to distort protein thiol redox state during lysis. However, measuring any molecule, even *in vivo*, can change the system in accordance with the Heisenberg principle. As Paulsen and Carroll remark [19], lysing cells exposes thiols to atmospheric ground state molecular dioxygen (O_2_). Exposure to 21% O_2_ coupled to transition metal ion availability, could result in artificial protein thiol oxidation likely via free radical mechanisms able to outcompete NEM labelling. For example, molecules (e.g., dopamine [154,155,156]) autoxidized to superoxide via a transition metal catalyst could lead to hydroxyl radical (^•^OH) production secondary to H_2_O_2_/metal mediated Fenton chemistry (e.g., Fe^2+^ + H_2_O_2_ → ^•^OH + ^−^OH). While Fenton chemistry could be mitigated by adding chelators, the reaction between ^•^OH and cysteine (*k* = 7.9 × 10^9^ M^−1^ s^−1^) is orders of magnitude faster than NEM mediated alkylation [74]. Lysis could lead to SOH loss via condensation with a reduced thiol to form a disulfide bond (i.e., SOH + RSH → RSSR + H_2_O), which occurs significantly faster (*k* ~10^5^ M^−1^ s^−1^) than alkylation [41]. Unless the local environment stabilized SOH, condensation would likely occur in vivo. For cases when SOH is stabilized (e.g., a binding protein can stabilize SOH in ORP1 [157]), condensation could confound reversible oxidation type and occupancy analysis. For example, condensation with GSH, present at ~10 mM in most cells [158], could be favored ex vivo leading to the misassignment of SOH as RSSG. Even in the presence of alkylating agents, the possibility of lysis induced *ex vivo* oxidation represents a perennial concern. 

### 3.5. Solutions

**Figure 6:**
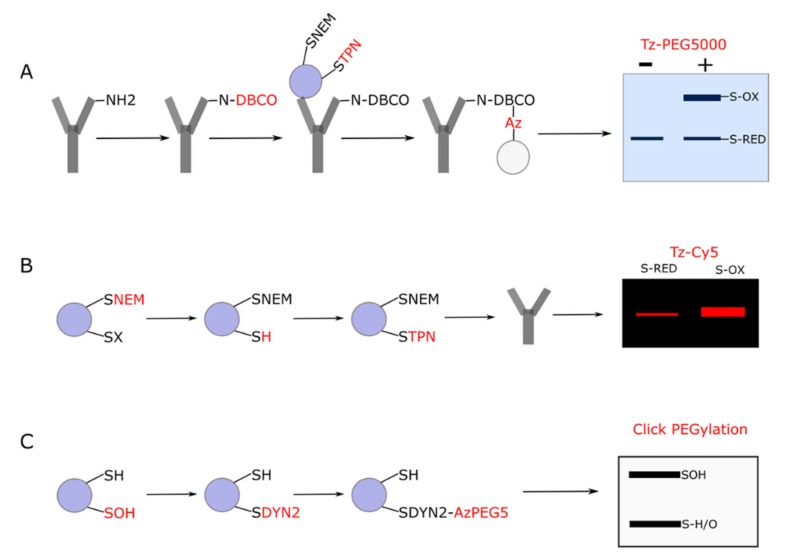
Novel, clickable immunological approaches to assess protein thiol redox state. (**A**). Antibody first Click PEGylation. Dibenzocyclooctyne (DBCO) functionalized *N*-hydroxysuccinimide is used to label NH_2_ moieties in the primary antibody. The DBCO labelled primary antibody is incubated with the sample to capture the TPN labelled target. Azide functionalized resin is used to capture the antibody-target complex via SPAAC. After stringently washing the captured complex via spin cups (omitted for clarity), the eluted target is reacted with Tz-PEG5 via IEDDA and mass shifts are visualized in gel via Coomassie staining. (**B**). Fluorescent IEDDA. After alkylating reduced thiols with NEM, reversibly oxidized thiols are reduced with TCEP before TPN labelling. The TPN labelled target is captured immunologically (as above) before the eluted target is conjugated with 6-methyltetrazine functionalized Cy5 (Tz-Cy5). Fluorescence is visualized in gel at the appropriate excitation and emission. Comparative parallel reduced and reversibly oxidized target fluorescence is depicted. (**C**). Reaction based target oxidative modification type profiling. The example shown considers SOH. SOH moieties are selectively labelled with Dyn-2 (SOH reactive warhead with a clickable alkyne) before being reacted with Azide functionalized PEG5000 via SPACC. Total SOH occupancy is then quantified via Click PEGylation. If Click PEGylation failed to detect a given target, then workflow A or B could be used.

The potential of the bulky PEG moiety to occlude antibody binding can be overcome by adopting an “antibody first” immunocapture workflow (Figure 6A). To do so, additional clickable immunocapture steps precede Tz-PEG5 labelling. Dibenzocyclooctyne (DBCO) functionalized *N*-hydroxysuccinimide is used to label the primary antibody by forming stable amide bonds with primary amines. The labelled antibody is added to the sample before being captured with an azide functionalized resin via strain-promoted azide–alkyne cycloaddition (SPAAC) [115]. The solid support and stable triazole enable the antibody-target complex to be isolated with high stringency and for the target to be eluted without the antibody, respectively. Following elution, Tz-PEG5 is added to initiate IEDDA and resultant mass shifts are visualized in gel via Coomassie or Silver Staining. Optionally, native polyacrylamide gel electrophoresis could be used to prevent SDS distorting mass shifted bands [144]. In gel analysis eliminates differential PEGylated protein transfer. Antibody first workflows represent a promising alternative to canonical Click PEGylation, especially when they can be readily coupled to selective reduction workflows. 

Even antibody first workflows may fail to adequately resolve relatively large proteins with many solvent exposed protein thiols. For example, the ~500 kDa ryanodine receptor 1 (RYR1) possesses over 100 thiols [159,160], which presents axiomatic difficulties for Click PEGylation. For proteins like RYR1, one could assess the weighted mean of all solvent exposed protein thiols in gel using the antibody first workflow to capture the target and substituting Tz-PEG5 for a 6-methyltetrazine functionalized fluorophore (e.g., Tz-Cy5); which could be quantified provided an equal amount of protein was loaded (Figure 6B). Alternatively, the fluorescent signal could be expressed relative to the total band after Coomassie or Silver staining. Importantly, antibody-first fluorescent workflows afford a useful tool to verify Click PEGylation findings, especially when reduced and reversibly oxidized protein thiol analysis is performed in parallel. 

Beyond affording a simple method to check successful Click PEGylation, anti-PEG makes it possible to use a single antibody to assess global RSSG, RSNO, and RSSR using selective reduction strategies. Single antibody global blotting is a useful, inexpensive tool to screen reversible thiol oxidation type. Single antibody reversibly modification type screening would be advanced by the development of a Tz-PEG20 reagent to reduce band complexity. 6-methyltetrazine functionalized Biotin is, however, commercially available and can be coupled to streptavidin functionalized fluorophores [137]. Optionally, immobile anti-PEG or streptavidin supports could be used to purify a certain reversible thiol oxidation type for proteomic analysis. It is, however, stressed that excellent technologies like biotinylated NEM already exist to use one tool (i.e., a streptavidin conjugated fluorophore) to assess multiple chemotypes at the global level. 

Opportunities exist to solve the alkylating agent SOH reactivity issue. Cochemé and colleagues [98] propose that: SOH could be specifically labelled with Dyn-2 (Figure 6C). Carroll’s group developed Dyn-2—a dual functional reagent containing a SOH reactive dimedone moiety coupled to a clickable alkyne group [150]. The expanded palette of carbon nucleophiles with enhanced SOH reactivity [161], set the stage to develop novel clickable tools to assess SOH using Click PEGylation. Furthermore, Fox’ group [162] have developed SOH reactive TCO, which enables Tz quenching to avoid ex vivo artefacts; such tools could be modified to enable IEDDA mediated Click PEGylation. Novel DiaAlk reagents afford similar opportunities to selectively assess SO_2_ [147]. Importantly, in vivo fluorescent SOH imaging compared to pan-SOH blotting enables one to gauge whether destructive lysis confounds the analysis. Cell permeable labelling reagents afford a means to label reduced thiols before lysis (mainly in the cytosol unless they are organelle targeted). However, the ability of TCO/Tz functionalized reagents to permeate the cell is unclear and should be tested before live cell labelling is undertaken. The development of SH/S^-^ selective tools coupled to reagents to directly probe each modification type without reduction by forming a diagnostic product allied to carefully buffer preparation should make it possible to advance redox proteomic and immunological analysis [153]; especially for contexts when only destructive analysis is currently possible (e.g., human skeletal muscle). At present, many reaction based technologies (e.g., a mutant glutathione synthase able to incorporate azide functionalized alanine instead of glycine into GSH to study RSSG [163]) would need to be modified (e.g., synthesis of TCO alanine) to be compatible with IEDDA Click PEGylation. Likewise, IEDDA compatible triarylphosphine ester [164] tools for RNSO would need to be synthesized. 

## 4. Opportunities: How to Use Click PEGylation to Advance Knowledge of Redox Biology

We define three opportunities to highlight how Click PEGylation can be used to advance knowledge of redox biology. First, Click PEGylation could be used to develop site-specific sentinels of mitochondrial superoxide production. Unravelling the providence of the superoxide detected by fluorescent reporters (e.g., MitoNeoD [165]) is challenging [166,167,168]. It is imperative to overcome existing challenges to rationally manipulate mitochondrial superoxide production to treat disease. Selective inhibitors of superoxide production at complex I and complex III make it possible to infer providence by assessing the change in superoxide reporter signal with and without the inhibitor [169,170]. While coupling selective inhibitors to a superoxide reporter is useful in cells, it is difficult to apply to tissues (e.g., due to their optical inaccessibility) and impossible to apply to humans until the inhibitors satisfy legislative requirements. Site-selective superoxide reporters would, therefore, advance the field. Using redox proteomics, Dröse group [171] have identified distinct subsets of thiols are reversibly oxidized by complex III, complex I forward, and complex I reverse electron transfer mediated superoxide production. Their findings raise the possibility of assessing site-specific superoxide production sentinels using Click PEGylation, provided subsequent works affirms their fidelity (i.e., they must faithfully respond to a single site out of over 15 known mitochondrial sites [172]). Assessing a panel of site-specific superoxide production sentinels using Click PEGylation could be used to translate literature suggesting hypoxia increases complex III mediated superoxide production to humans [173,174,175]. Such work would advance understanding of how we sense O_2_ [176]. 

Second, Click PEGylation could be used to assess exercise-induced redox signaling [177,178,179,180,181,182,183,184]. A recent comprehensive review [185] concluded that redox signaling is central to exercise responses and adaptations (e.g., mitochondrial biogenesis [186,187]). Knowledge of exercise induced redox signaling in human skeletal muscle is, however, fragmentary [188,189]. Opportunities exist to decipher exercise induced redox signals in human skeletal muscle using integrative unbiased redox proteomics, Click PEGylation and MRM. We propose using skeletal muscle biopsies to: (1) identify reversibly oxidized proteins using unbiased redox proteomics; (2) to quantify candidate reversibly oxidized proteins using Click PEGylation; (3) identify and quantify modified thiols using MRM [90,190,191]; and (4) interrogate reversible thiol oxidation type or assess function (e.g., enzyme assays with and without TCEP/DTT). Optionally, Click PEGylated lysates could be used to assess proteins that are difficult to detect by mass spectrometry (e.g., KEAP1) owing to the abundance of contractile proteins (e.g., myosin) [192]. Moreover, parallel in vitro skeletal muscle cell exercise models with and without site directed Crisper-Cas9 [193,194] thiol mutagenesis could be used to determine the biological significance of reversible thiol oxidation using exercise reporters (e.g., mitochondrial mass), provided the thiol was non-catalytic. Mutating a catalytic thiol (e.g., mutating Cys215 in PTB1B [195]) presents obvious difficulties for functional studies [38]. As a murine study attests [196], the integrated approach proposed would yield unprecedent insight into exercise induced redox signaling in humans [197].

Third, Click PEGylation could help disambiguate the molecular basis of oxidative stress in assisted reproduction technologies like in vitro fertilization (IVF) [198,199,200]. IVF induced oxidative stress stems from exposing oocytes/zygotes to 21% O_2_; which is exacerbated by culture conditions (e.g., nutritional antioxidant deficiency, ambient light exposure, and transition metal catalyzed autoxidation [201,202]). The molecular basis of oxidative stress in IVF is unclear. The role of reversible thiol oxidation is unconsidered. Considering reversible thiol oxidation using Click PEGylation could shift the paradigm from oxidative macromolecule damage (e.g., lipid peroxidation) alone as a driver of oxidative stress to disrupted redox signaling manifested by aberrant reversible thiol oxidation. IVF could increase fractional reversible thiol oxidation of strategically important thiols leading to impaired fertility secondary to dysregulated redox signaling; especially if the pre-existing infertility distorted the thiol proteome. For example, the early cell cycle is regulated by redox sensitive protein phosphatases (e.g., cdc25c [203]) [204]. Increased fractional reversible thiol occupancy and/or shift towards SO_2_/SO_3_ occupancy of protein phosphatases could arrest the early cell cycle setting the stage for apoptotic/necrotic mediated embryo fragmentation. Reversing increased fractional thiol oxidation occupancy may be constrained by the functionally immaturity of the glutathione and thioredoxin dependent enzyme systems [205,206]. Even if they were fully operational NADPH synthesis could be limiting [207]. From a clinical perspective, redox proteomics could be used to identify reversibly oxidized proteins secreted by viable and non-viable embryos to develop non-invasive molecular biomarkers assessed using immunological techniques to select the “best” embryos for IVF. Immunological techniques to assess the redox state of proteins secreted in the picomolar and femtomolar range would, however, be required. Until then, Click PEGylation could be used to explore a role for disrupted redox signaling in IVF. 

## 5. A Concluding Perspective and Recommendations

Most biologically relevant reactive species appear and disappear on the nanosecond timescale. For instance, the lifetime of even chemically constrained species like superoxide [208] is limited by a set of kinetically efficient reactions (e.g., superoxide dismutase isoform catalyzed dismutation to O_2_ and H_2_O_2_ [208,209,210]). The analytical challenges of measuring reactive species directly are, therefore, considerable [211,212,213,214]. While significant analytical challenges have been surmounted (e.g., real time ratio-metric H_2_O_2_ and GSH measurement [215,216,217,218,219]), it is essential to measure what reactive species are doing by chemically foot-printing their biological reactivity. For many years, the scope of chemical foot-printing was narrowed by oxidative stress being viewed as solely deleterious and a resultant focus on measuring oxidative macromolecule adducts. Measuring many oxidative macromolecule adducts is prone to artefact [220]; and, moreover, unsuitable when one is interested in redox signaling [221], which is often orthogonal to oxidative macromolecule damage [222]. We contend the centrality of the heterogenous thiol proteome to oxidative stress, antioxidant defense, and redox signaling provides a conceptual mandate to reimagine chemical foot-printing. Specifically, the thiol proteome is an endogenous reactive species sensor that dynamically transforms labile signals into relatively stable sulfur oxidation signatures (i.e., chemical footprints) capable of enacting diverse biological outcomes by altering protein function. Measurement and function can coalesce into a single entity when one foot-prints the chemical biology of reactive species and indeed key redox enzymes (recall redox enzymes bidirectionally control oxidation state) by assessing protein thiol redox state. That is to say, assessing protein thiol redox state is as important as measuring reactive species directly. Considering many overtly flawed assays (e.g., DCF, see [223,224]) still plague the field, it is surprising that Click PEGylation—a technically sound assay—has seldom been used to interrogate the functional interface between reactive species and the thiol proteome. By continually refining Click PEGylation and complementary redox proteomic approaches, we believe it is possible to harness immunological techniques to significantly advance current knowledge. To do so, we provide a concluding set of Click PEGylation recommendations:

1. Identify the number and solvent exposure (if structural models allow) of the target protein thiol, as well as, protein mass to rationally select the PEG size and gel percentage. Note some thiols can selectively become exposed to solvent (termed cryptic thiols).
2. Select polyclonal antibodies if possible or monoclonal antibodies distal to the PEGylated thiols.
3. Carefully prepare reaction buffers (e.g., pH in lysis buffer and SDS in Laemmli buffer) and consider using TCEP as a generic reductant to avoid sulfur autoxidation artefacts.
4. Include an unreacted control with and without DTT in the Laemmli buffer to assess target recognition and RSSR mediated oligomeric mobility shifts, respectively.
5. Consider direct reaction strategies to assess SOH occupancy.
6. Judiciously appraise the merits of selective reduction strategies (they can affect other modification types).
7. Perform reciprocal reduced and reversibly oxidized Click PEGylation analysis.
8. If reciprocal reduced and reversibly oxidized Click PEGylation implies SO_2_/SO_3_ occupancy, then consider direct reaction strategies or mass shifting reduced and reversibly oxidized proteins together to confirm. Optionally, use a recombinant sulfiredoxin reduction system.
9. Use complementary redox proteomics (e.g., MRM) and functional assays (e.g., enzyme assay) if possible.
10. If Click PEGylation fails, consider antibody first workflows.

## Figures and Tables

**Table 2:** Key advantages and disadvantages of Click PEGylation. Modified with permission from Cobley and colleagues [116].

Advantage	Disadvantage
Catalyst-free, kinetically efficient, and bio-orthogonal Inverse Electron Demand Diels Alder (IEDDA).	The ability of PEG to sterically occlude antibody binding.
Able to interrogate hypothesis driven biological questions.	SDS-PEG can interact to distort the bands.
Able to quantify reversible thiol oxidation occupancy.	Multiple bands preclude multiplexing.
Able to quantify the relative contribution of each modified thiol relative to total reversible oxidation.	In many cases, MRM is required to identify the thiols modified.
Discloses the site (s) modified for proteins with a single thiol or when each thiol has been mass shifted.	Potential for inefficient transfer of PEGylated proteins.
Compatible with selective reduction strategies to identify and quantify reversible oxidation type.	Careful PEG, antibody and gel size selection is required.
Compatible with direct chemical reaction analysis (e.g., Dyn-2 for SOH).	Destructive analysis can introduce lysis artefacts.
Compatible with organelle, whole-cell, and tissue lysate analysis.	Investigator bias when used to interrogate a hypothesis.
Harnesses standard equipment and techniques (e.g., Western blotting).	Unsuitable for certain species due to the lack of appropriate antibodies.
Uncomplicated and rapid data analysis.	Unsuitable for large proteins with many solvent exposed thiols.
Internal normalization obviates the need for a loading control.	Requirement for µg/ml protein for Western blotting in complex samples *.
Suitable as an orthogonal workflow to redox proteomics.	Identifying and quantifying SO_2_/SO_3_ occupancy is challenging.
Suitable for hydrophobic and difficult to digest proteins.	Snapshot analysis limits temporal resolution.
Flexible—can readily be adapted (e.g., antibody-first workflows).	

* ng/mL may be possible with sample purification.
